# Bioaerosol-induced in vitro activation of toll-like receptors and inflammatory biomarker expression in waste workers

**DOI:** 10.1007/s00420-023-01984-7

**Published:** 2023-05-27

**Authors:** Elke Eriksen, Anani Komlavi Afanou, Anne Straumfors, Pål Graff

**Affiliations:** STAMI, National Institute of Occupational Health, Gydas Vei 8, 0363 Oslo, Norway

**Keywords:** Occupational exposure, Bioaerosols, TLR2, TLR4, Biomarkers, Health effects, Waste sorting, Symptoms

## Abstract

**Purpose:**

Occupational exposure to bioaerosols during waste handling remains a health concern for exposed workers. However, exposure-related health effects and underlying immunological mechanisms are still poorly described.

**Methods:**

The present study assessed the inflammatory potential of work-air samples (*n *= 56) in vitro and investigated biomarker expression in exposed workers (*n* = 69) compared to unexposed controls (*n* = 25). These quantitative results were compared to self-reported health conditions.

**Results:**

Personal air samples provoked an activation of TLR2 and TLR4 HEK reporter cells in one-third of all samples, indicating that the work environment contained ligands capable of inducing an immune response in vitro*.* Monocyte levels, as well as plasma biomarker levels, such as IL-1Ra, IL-18 and TNFα were significantly higher in exposed workers, compared to the control group when confounding factors such as BMI, sex, age and smoking habits were accounted for. Furthermore, a significant exposure-related increase in midweek IL-8 levels was measured among exposed workers. Tendencies of increased prevalence of health effects of the respiratory tract were identified in exposed workers.

**Conclusion:**

Inhalable dust provoked TLR activation in vitro, indicating that an exposure-related immune response may be expected in susceptible workers. However, despite significant differences in inflammatory plasma biomarker levels between exposed and unexposed workers, prevalence of self-reported health effects did not differ between the groups. This may be due to the healthy worker effect, or other factors such as adequate use of personal protective respiratory devices or adaptation to the work environment with reduced activation of the immune system.

**Supplementary Information:**

The online version contains supplementary material available at 10.1007/s00420-023-01984-7.

## Introduction

National waste management strategies are greatly affected by the global shift towards greener societies and the sustainable use of natural resources. Additionally, the introduction of new waste sorting technologies in combination with an increase in per capita produced waste demands higher manpower in the waste industry (NOA 2021; Statistics Norway 2022). Waste collecting, processing and sorting are physically demanding work tasks. In addition to the physical strain, occupational exposure to a heterogenous mixture of inhalable bioaerosols contains organic material as well as particles of microbial origin (Eriksen et al. 2023; Madsen et al. 2021); vapours and gasses increase the burden on workers’ health and wellbeing. Exposure to varying levels of organic dust that contained microbial components has reportedly caused elevated incidence rates of adverse respiratory health effects (Eriksen et al. 2023; Straumfors et al. 2016; Vimercati et al. 2016), the gastrointestinal system (Ivens et al. 1999), septic shock (Martin 1999), as well as dermatological symptoms (Megna et al. 2017). Bioaerosol exposure during waste handling is of high relevance in terms of work risk assessment, as the contaminants may have synergetic or additive effects on the workers’ immune system.

The innate immune system forms the first line of defence against external stimuli with potential toxicological outcomes. The human respiratory system is equipped with multiple physical, chemical and cellular defence mechanisms to prevent pulmonary cell injuries and tissue damagev. Among these, toll-like receptors (TLR) that recognize a wide array of evolutionary conserved pathogen-associated molecular patterns (PAMP) are important modulators of an immune response provoked by invading microbial epitopes, such as bacterial lipopolysaccharides (LPS) and lipoteichoic acid (LTA). Cell models have readily been used in occupational toxicity studies to investigate the effects of work environmental samples in vitro*.* Both LPS and LTA have been shown to be immunostimulatory by activating the nuclear factor-kappa B (NFkB) pathway that results in the transcription of inflammatory genes that initiate the synthesis of inflammatory signalling molecules, such as cytokines, chemokines and endothelial adhesion molecules (Eriksen et al. 2022; Idriss and Naismith 2000; Long et al. 2009; Melotti et al. 2001; Yoshimura et al. 1997). A rapid increase in cytokine levels can cause systemic effects that may be experienced as flu-like symptoms (Descotes and Vial 2007) which have been reported in correlation with exposure to high levels of organic dust (Sandström et al. 1992; Thorn et al., 1998; Von Essen et al. 1990). Most cytokines and chemokines, however, are short lived and primarily exert their effect within hours of secretion (Monastero and Pentyala 2017). Furthermore, it has been proposed that differences in biomarker levels may be influenced by genetic polymorphisms, lifestyle, BMI, sex and smoking habits (de Torres et al. 2011; Leng et al. 2004; Timpson et al. 2011; Wegner et al. 2017). Nevertheless, cytokines have the potential to be used as biomarkers to investigate acute exposure-related health effects (Heldal et al. 2016; Wikuats et al. 2022).

The present study aimed on gaining knowledge on potential occupational exposure-related health effects in waste workers, by investigating the TLR activation potential of inhalable dust in vitro and levels of general and lung-specific biomarkers in exposed workers in contrast to an unexposed control group.

## Material and methods

### Study population

A total of 5 female and 65 male waste workers (exposed group) and 8 female and 17 male office personnel (control group) from four different waste sorting plants (plant A:D) participated in the study. The average employment time at the current workplace among exposed workers was 2.6 years (range 1 month – 13 years), whereas among controls it was on average 3.6 years, ranging from 1 month to 14 years. Data on previous employment were not collected in the present study. Participation in the study was voluntary, and informed consent was obtained prior to participation. Each participant answered a survey containing personal, occupational and health-directed questions. Work-air samples of total dust, endotoxins and microbial DNA were collected as previously described (Eriksen et al. 2023). The study was approved by the Regional Ethics Committee in Oslo, REC South-East B (Ref.No.: 34312).

### Air-samples: collection, analysis & in vitro exposure experiments

Air samples were collected at six waste sorting plants (plants A: F). Personal full-shift air samples (mean sampling time 6.9 h) were collected using antistatic polypropylene filter cassettes (TeknolabAS, Norway) containing 37-mm hydrophilic polycarbonate filters (pore size of 0.8 µm, Merck Millipore KgaA, Germany). Filter cassettes were connected to an air-pump (GS5200, GSA Messgerätebau GmbH, Germany) and operated at an air flow of 2.0 l/min (± 10%). Unexposed filters were included as controls for each 10^th^ field sample. Exposed filters were transferred to sterile 15-ml tubes under aseptic conditions. Dust was eluted in 5 ml PBS-BSA 0.1% by sonication for 5 min at room temperature (RT) followed by orbital shaking at 500 rpm for 60 min. Subsequently, filters were removed with sterile tweezers and dust suspensions were aliquoted and stored at −80 °C until in vitro experiments were conducted.

#### In vitro experiments

Human embryonic kidney (HEK) 293 cell lines (Invivogen, France) that were transfected with TLR2 and TLR4 inducible reporter genes, as well as a parental HEK 293 TLR null cell line (Invivogen, France), were exposed to work environmental air samples collected at all participating plants, following the procedures described by Brummelman et al. (2015), however, with some minor modifications. Cells were cultured for two passages before they were split on sterile Nunclon flat bottom microplates (Nunc Edge 96-Well, Thermo Fisher). In the exposure experiments, each well contained 180 μL cell suspension with cell concentration of 2.8 × 10^5^ cells/ml. The cells were grown in Dulbecco’s Modified Eagle Medium (DMEM)—Glutamax with high glucose content and supplemented with 10% inactivated Foetal Bovine Serum (FBS) and HEK Blue selection antibiotics as recommended by the manufacturer to ensure adequate growth conditions. In the exposure experiments, cells were first incubated for 3 h at 37 °C, high humidity and 5% CO_2_, before 20 µl of dust suspension was added to each well and the cells were incubated further for 22 h. Twenty microliters of the cell supernatant were then transferred to fresh 96-well plates and treated with 180 µl Quanti Blue solution (Invivogen, France). Plates were incubated for another 180 min before colour development was measured spectrophotometrically at a wavelength of 649 nm using a BioTek Synergy Neo2 hybrid multimode reader (Agilent Technologies, USA). Ultrapure LPS (1 µl/ml), LTA (1 µl/ml), Zymosan (10 µl/ml), as well as PBS-BSA (10x), PBS and endotoxin free water were included as positive and negative controls. Exposure experiments were conducted over a period of two weeks to keep the number of cell line passages to a necessary minimum. All experiments were repeated once and run as biological parallels. SEAP absorbance levels in the cell supernatant after exposure were reported as mean of biological parallels corrected for absorbance of the unexposed samples’ background levels.

### Blood samples: collection and analysis

Blood samples were collected at plant A: D. A total of 5 females and 16 males in the control group, and from 3 females and 48 males in the exposed group prior to shift on the first and third workday of the week using EDTA (BD Vacutainer K2E, BD, US) and Serum-tubes (BD Vacutainer SST II Advance, BD, US) for plasma and serum separation, respectively. The tubes were inverted several times after sampling. EDTA tubes were centrifuged for 12 min at 1500 g to separate plasma from blood cells. Serum tubes (Gel tubes) were allowed to coagulate for 30 min at room temperature and then centrifuged at 1500 g for 12 min. Plasma and serum samples were aliquoted and stored at −80 °C until further analysis. Each one serum and full blood sample were sent to an accredited laboratory (Fürst Norway) for analysis of CRP and blood leukocyte levels within 24 h after sampling. Results were reported as individually measured levels in accordance with the laboratory’s reference range (Table S5). Samples from one individual from the control group were removed from further analyses as the worker was self-reportedly ill on both sampling days.

### LUMINEX

Plasma levels of biomarkers were analysed using two different multiplex kits. The first kit, a custom multiplex human cytokine assay included the following analytes: IL-1α, IL-1β, IL-1ra, IL-2, IL-4, IL-6, IL-8, IL-10, IL-12(p70), IL-13, IFN-γ, TNF-α, GM-CFS (Bio-Plex, BioRad Laboratories Inc., Norway), whereas the second kit, a multiplex human magnetic Luminex assay included CCL2, ICAM-1, IL-2, IL-18, MMP-12, S100B, TGF-α, CD40 ligand, IL-1 β, IL-17, IL-33, procalcitonin and SP-D (R&D Systems Inc., MN, USA). Plasma samples diluted 1:4 (BioRad) and 1:2 (R&D), and the assays were applied as recommended by the manufacturers and run as parallels. Analyses were performed on a Bio-Plex MAGPIX Multiplex reader (Bio-Rad Laboratories, Inc., CA, US). Plasma levels of analytes were estimated based on an eight-star (BioRad) and six-star (R&D) standard reference curve (Table S9). Positive and negative controls as well as blank samples were included in each experiment. Levels of biomarkers with respiratory relevance were reported as observed concentrations in pg/mL within 80–120% of the respective standard curve. Individual levels were to a large extent below the level of detection (LOD, Table S9) for 14 of the 24 investigated biomarkers. Thus, biomarkers with a minimum of 60% of individual measurements above the LOD were included in the analyses: IL-8 (68%), TNFα (82%), IL-1Ra (78%), CCL2 (100%), ICAM1 (100%), IL-18 (100%), S100B (71%), procalcitonin (100%) and SP-D (100%). For these analytes, levels below the LOD were randomly replaced with levels ranging from LOD to LOD/2.

### Statistical analyses

All data analyses were performed in R/Rstudio (R version 4.2.2/ Rstudio version 2022.12.0) using ggplot2 (Wickham 2016) for data visualization and the rstatix package (Kassambara 2021), the stats package (R Core Team 2021) and the lme4 package (Bates et al. 2015) for statistical analysis. Data were not normally distributed (Shapiro–Wilk’s normality test, *p*-value < 0.05 for statistical significance) and were therefore log-transformed prior to analyses. A paired-sample t-test was used to identify significant differences (*p*-value < 0.05) in TLR activation between cell types. Differences in symptom prevalence between exposure groups were investigated based on a chi square test.

A linear mixed effect model (lmer) was applied to investigate the effect of the exposure group (exposed vs. unexposed controls), day, BMI, age, sex and smoking habits on the blood markers. Repeated measurements per individual were included as random effect (person id).

1 $$Log\left( {analyte} \right)\, = \,Intercept\, + \,\exp osure \, group\, + \,day\, + \,BMI\, + \,age\, + \,sex\, + \,smoking\, + \,\left( {1|person} \right)\, + \varepsilon$$

A linear model was used to estimate the effect of exposure to total dust, endotoxins and microbial DNA concentrations on TLR activation patterns.

2 $$Log\left( {SEAP} \right)\, = \,\log \left( {\exp osure \, measurement} \right)\, + \,\varepsilon$$

A linear model accounting for BMI, age, sex and smoking habits was used to estimate the effect of exposure levels on midweek blood biomarker levels.

3 $$Log\left( {level} \right)\, = \,\log \left( {\exp osure \, measurement} \right)\, + \,BMI\, + \,age\, + \,sex\, + \,smoking\, + \varepsilon$$

## Results

### Study population–demographics, nicotine habits and general health

**Fig. 1:**
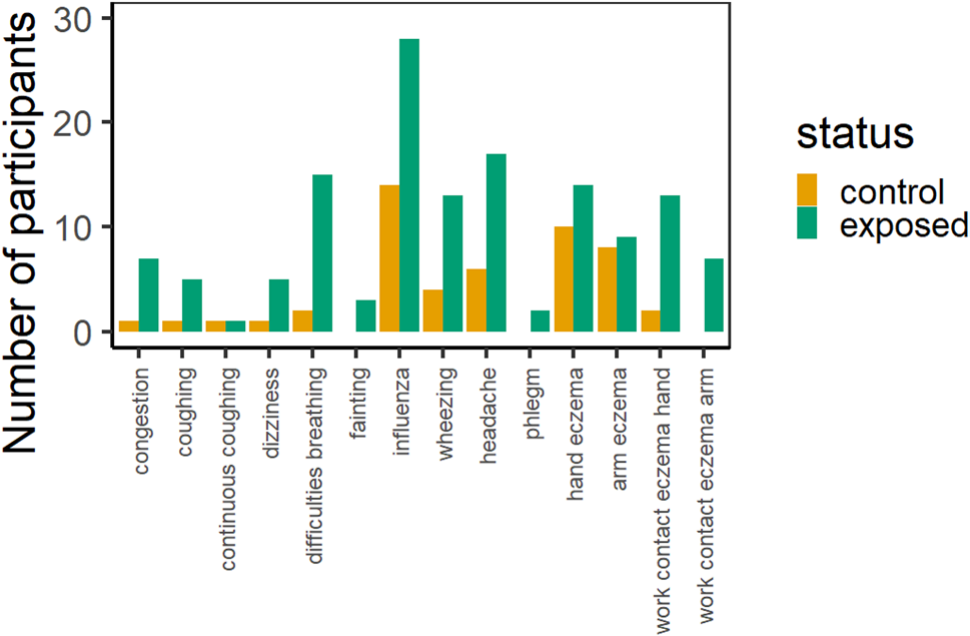
Self-reported symptom frequencies in exposed workers and unexposed control group

The average age of exposed female and male workers was 29 and 39 years, respectively, whereas female and male controls were on average 36 and 45 years of age (Table 1). The average BMI among male participants was 27 in both groups, whereas the average BMI in exposed females (BMI = 22) was somewhat lower compared to females in the control group (BMI = 25). Among exposed workers, 1 female and 15 (23%) males were active smokers, whereas no females and 5 (29%) males in the control group regularly smoked. The proportion of past smokers was higher in the control group (female: 25%, male: 53%) compared to the exposed group (female: 0%, male: 34%). Participants in the control group used significantly more often prescription drugs (*p*-value < 0.001). The prevalence of allergies was twice as high among men in the control group compared to the exposed population, and vice versa for female participants. Self-reported symptom frequencies were tendentially, higher in exposed workers (Fig. 1); however, differences were statistically not significant.

**Table 1:** Demographics study population

Sex	Exposed		Unexposed controls	
	Female (*n* = 4)	Male (*n* = 64)	Female (*n* = 8)	Male (*n* = 17)
Mean age (range) years	29 (21–40)	39 (20–65)	36 (29–41)	45 (41–55)
Mean height (range) meters	1.68 (1.61–1.74)	1.79 (1.65–2.03)	1.73 (1.63–1.80)	1.81 (1.66–2.03)
Mean weight (range) kg	62 (60–68)	88 (65–130)	76 (58–90)	87 (56–110)
Mean BMI (range)	22 (20–24)	27 (20–41)	25 (22–31)	27 (20–31)
Past smokers (%)	0	22 (34)	2 (25)	9 (53)
Active smokers (%)	1 (20)	15 (23)	0	5 (29)
Snus users (%)	0	20 (31)	1 (13)	1 (6)
Prescription drugs (%)	1 (20)	14 (22)	4 (50)	13 (76)
Allergies (%)	3 (60)	14 (22)	3 (38)	8 (47)
Underlying disease^1^ (%)	0	1 (2)	0	2 (12)
Doctor diagnosed asthma (%)	0	8 (13)	0	1 (6)

^1^Participants who had a doctor diagnosed disease other than asthma.

### In vitro experiments

**Fig. 2:**
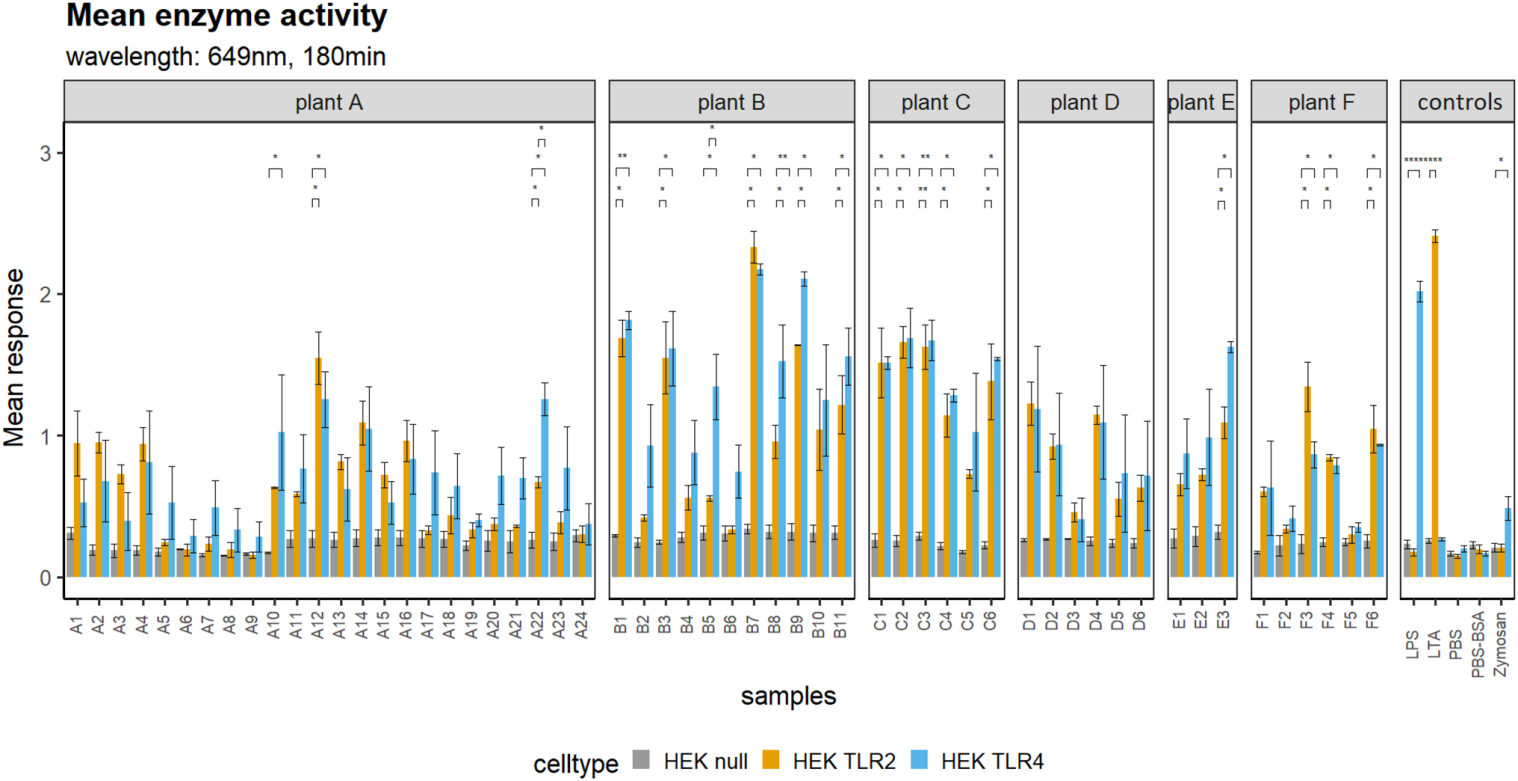
TLR activation patterns by sample and waste sorting plant (plant A: F). Positive (LPS, LTA, Zymosan) and negative (PBS, PBS-BSA) controls in right panel. Absorbance levels in HEK null cells (grey), HEK TLR2 cells (orange), HEK TLR4 cells (blue). Significant differences between TLR transfected cells and the null cell line indicated with asterisk. Standard error included as error bars

**Fig. 3:**
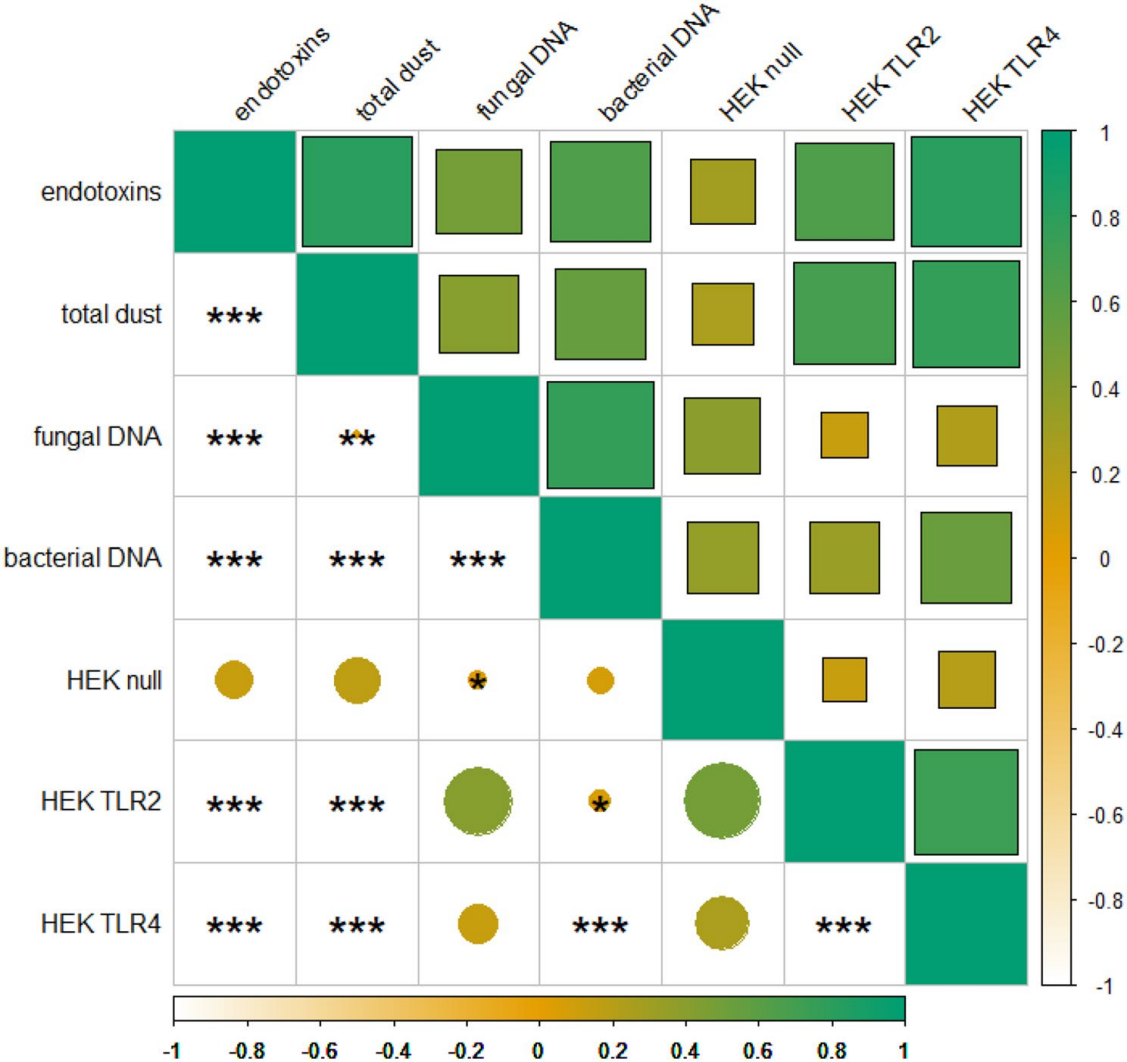
Correlation matrix (Pearson correlation coefficients, upper half) and Benjamini–Hochberg corrected p-values (lower half) for background corrected TLR activation levels and exposure measurements in plant A: F

A total of 56 personal air filter samples were subjected to in vitro experiments. Significant activation (*p*-value < 0.05) of TLR2 by stock concentration was measured in 30% of all samples, whereas 33% of the samples significantly activated TLR4 (Fig. 2). Significant differences in activation patterns between TLR2 and TLR4 were observed in two samples. TLR 2 and TLR 4 activation patterns correlated significantly (*p*-value < 0.001) and positively with endotoxin and total dust levels (Fig. 3). Significant (*p*-value < 0.001) positive correlation was also observed for TLR2 and TLR4 cells and bacterial DNA concentrations (Table S1). Parental null HEK cell lines showed no significant correlation with any of the assessed parameters.

**Fig. 4:**
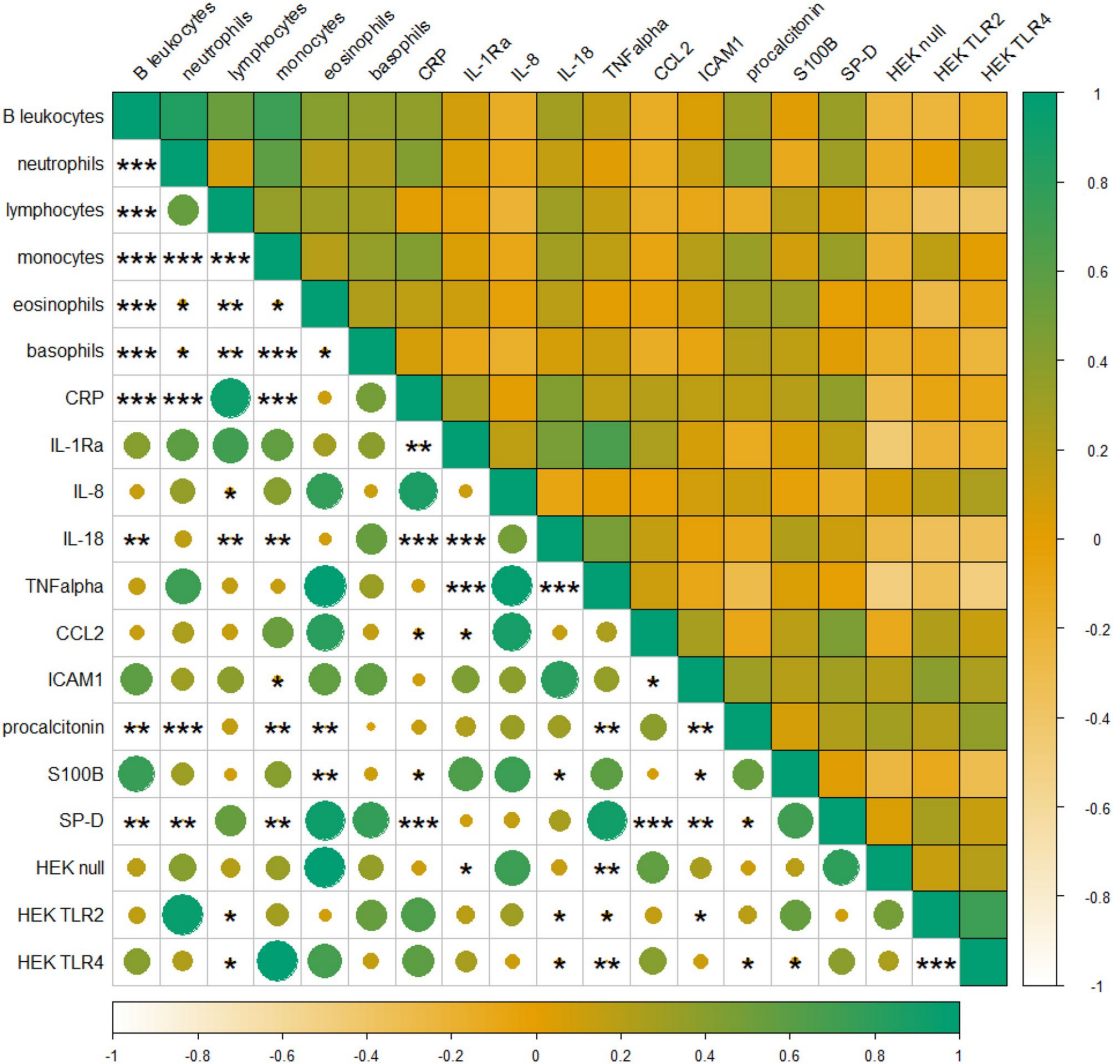
Correlation matrix (Pearson correlation coefficients, upper half) and Benjamini–Hochberg corrected p-values (lower half) of blood leukocytes and plasma biomarkers in exposed workers as well as dust-induced TLR activation in vitro from plant A: D

HEK activation patterns correlated positively and significantly to various blood leukocyte counts and plasma biomarkers. TRL2 cell activation patterns were significantly and positively associated to ICAM1 levels; however, negative correlations to lymphocyte levels as well as to IL-18 and TNFα were identified (Fig. 4). Elevated TLR4 activation levels correlated significantly and positively with procalcitonin levels. Significant negative correlations were identified for TLR4 activation and lymphocyte levels, as well as IL-18, TNFα and S100B.

### Plasma biomarker levels and exposure measurements

Plasma biomarker levels were in general comparable between the exposed and unexposed control group; however, significantly increased levels of IL-1Ra, IL-18 and TNFα were identified in the exposed group (Table 2). Procalcitonin levels were significantly associated to elevated BMI; the effects were, however, weak. CCL2, ICAM1 and procalcitonin levels were significantly higher in female participants, whereas CRP levels were significantly lower compared to males. Significantly increased IL-18 levels were associated to smoking. Correlation analysis between plasma biomarkers and blood leukocyte levels revealed significant and strong positive correlations, such as between CRP and neutrophils and monocytes, as well as between IL-18 and lymphocytes and monocytes in exposed workers (Fig. 4). Midweek exposure levels of total dust were significantly and negatively correlated to midweek TNF-α and IL-18 levels and positively to IL-8 levels, whereas endotoxin levels were significantly and positively associated to elevated plasma levels of ICAM-1 and negatively to TNF-α (Table S2).

**Table 2:** LMER model of plasma biomarker levels in waste workers

Predictors	IL-1Ra		IL-8		IL-18		TNFα		CCL2		ICAM1		Procalcitonin		S100B		SP-D		CRP	
	Estimates	*p *	Estimates	*p *	Estimates	*p *	Estimates	*p *	Estimates	*p *	Estimates	*p *	Estimates	*p *	Estimates	*p *	Estimates	*p *	Estimates	p
Intercept	0.716	0.582	−1.803	0.122	4.409	<0.001	0.361	0.815	4.179	<0.001	12.046	<0.001	2.356	<0.001	1.670	0.296	7.585	<0.001	−4.637	<0.001
workday (ref ‘Monday’)	0.113	0.512	0.010	0.970	−0.017	0.450	−0.046	0.861	0.003	0.886	−0.002	0.844	0.031	0.234	−0.089	0.582	−0.037	0.071	−0.075	0.208
exposure status (ref ‘control’)	0.864	0.036	−0.146	0.689	0.297	0.017	1.115	0.023	0.128	0.202	−0.003	0.983	−0.017	0.888	−0.505	0.315	0.182	0.139	0.393	0.078
BMI	0.052	0.327	−0.022	0.634	0.025	0.112	-0.056	0.370	0.011	0.412	0.020	0.250	0.033	0.035	0.031	0.634	0.020	0.216	0.148	<0.001
sex (ref ‘female’)	−0.642	0.321	0.206	0.721	−0.009	0.961	0.030	0.969	0.475	0.003	0.671	0.002	0.842	<0.001	1.403	0.078	0.200	0.303	−0.970	0.006
age	0.001	0.970	0.026	0.145	−0.000	0.973	0.013	0.598	−0.000	0.924	−0.004	0.563	−0.000	0.959	−0.006	0.792	0.008	0.184	0.031	0.005
smoking habits	0.867	0.051	−0.169	0.673	0.480	<0.001	0.820	0.121	−0.078	0.470	0.212	0.133	0.158	0.220	−0.212	0.695	0.051	0.699	0.208	0.385
Random Effects																				
σ2	0.89		2.07		0.02		2.07		0.02		0.00		0.02		0.78		0.01		0.10	
τ00	1.53 identifyer		0.48 identifyer		0.17 identifyer		1.69 identifyer		0.11 identifyer		0.21 identifyer		0.16 identifyer		2.60 identifyer		0.18 identifyer		0.54 identifyer	
ICC	0.63		0.19		0.92		0.45		0.87		0.98		0.89		0.77		0.94		0.84	
N	69 identifyer		69 identifyer		69 identifyer		69 identifyer		69 identifyer		69 identifyer		69 identifyer		69 identifyer		69 identifyer		69 identifyer	
Observations	128		128		128		128		128		128		128		128		128		127	
Marginal R2 / Conditional R2 *	0.090 / 0.665		0.028 / 0.211		0.217 / 0.935		0.082 / 0.495		0.210 / 0.899		0.219 / 0.983		0.370 / 0.930		0.062 / 0.783		0.152 / 0.945		0.414 / 0.905	

^*^Marginal R^2^ considers variance in fixed effects. Conditional R^2^ considers variance of fixed and random effects. The model investigated the effect of fixed factors, such as workday (Monday as reference), exposure status (unexposed controls as reference), *BMI* sex (female as reference), age and smoking habits on plasma biomarker levels. A random effect (identifier) allowed for variation in individual intercepts

### Blood leukocyte levels and exposure measurements

Blood levels were generally within the reference range or the respective analyte and did not vary between exposed and controls. However, the number of individuals with blood levels outside the reference range was higher among exposed workers compared to the control group. Monocyte counts were significantly higher in the exposed group (*p*-value < 0.001) (Table 3 & Figure S3). Elevated leukocyte, neutrophil and eosinophil levels were significantly correlated to elevated BMI, however, with weak effects. Furthermore, increased leukocyte levels were positively and significantly associated with smoking. An elevation in blood leukocyte levels correlated positively and significantly with various plasma biomarkers, such as between neutrophils and IL-18, procalcitonin and SP-D in exposed workers, whereas monocyte levels were significantly correlated to IL-18, ICAM1, procalcitonin and SP-D (Fig. 4). Exposure levels of total dust were significantly correlated to a slight decrease in midweek basophil levels in exposed workers (Table S2).

**Table 3:** LMER model of blood leucocyte levels in waste workers

Predictors	Blood leukocytes		Lymphocytes		Neutrophils		Monocytes		Eosinophils		Basophils	
	Estimates	p	Estimates	p	Estimates	p	Estimates	p	Estimates	p	Estimates	p
Intercept	1.290	< 0.001	0.643	0.011	0.444	0.086	−1.545	< 0.001	0.136	0.068	−0.007	0.872
workday (ref ‘Monday’)	−0.014	0.381	−0.052	0.026	0.034	0.249	−0.071	0.067	−0.018	0.020	0.012	0.088
exposure status (ref ‘control’)	0.090	0.114	0.042	0.595	0.095	0.240	0.276	< 0.001	−0.019	0.415	−0.019	0.153
BMI	0.022	0.004	0.017	0.085	0.023	0.031	0.015	0.115	0.008	0.007	0.003	0.120
sex (ref ‘female’)	−0.121	0.176	−0.191	0.124	−0.093	0.463	0.075	0.528	−0.041	0.265	−0.008	0.720
age	0.001	0.854	−0.006	0.129	0.004	0.266	0.005	0.155	−0.003	0.005	0.000	0.926
smoking habits	0.264	< 0.001	0.263	0.002	0.237	0.007	0.418	< 0.001	0.083	0.001	0.029	0.047
Random effects												
σ^2^	0.01		0.01		0.02		0.04		0.00		0.00	
τ_00_	0.03 _identifyer_		0.07 _identifyer_		0.06 _identifyer_		0.04 _identifyer_		0.01 _identifyer_		0.00 _identifyer_	
ICC	0.82		0.82		0.74		0.52		0.79		0.47	
N	69 _identifyer_		69 _identifyer_		69 _identifyer_		69 _identifyer_		69 _identifyer_		69 _identifyer_	
Observations	129		122		122		122		122		122	
Marginal R^2^ / Conditional R^2^ *	0.271 / 0.866		0.162 / 0.848		0.182 / 0.786		0.363 / 0.694		0.229 / 0.837		0.123 / 0.535	

^*^Marginal R^2^ considers variance in fixed effects. Conditional R^2^ considers variance of fixed and random effects. The model investigated the effect of fixed factors, such as workday (Monday as reference), exposed status (unexposed controls as reference), *BMI* sex (female as reference), age and smoking habits on blood leukocyte levels. A random effect (identifier) allowed for variation in individual intercepts

### Self-reported health effects

The health status of participants’ respiratory system was assessed as presence/absence of symptoms, such as asthma, difficulties breathing, congestion of airways, coughing and phlegm (Fig. 1). Among the 69 exposed workers, 14% suffered from doctor diagnosed asthma. Also, 25% of all exposed workers reported to regularly have difficulties breathing, 9% congestion of airways, 8% coughing and 5% phlegm. Among the 25 controls, 3% suffered from doctor diagnosed asthma, 11% reported difficulties breathing, 3% congested airways and 5% coughing (Table 1). Phlegm was not reported in the control group. No significant differences in symptom frequencies were observed between the two groups.

## Discussion

This study investigated potential immunological effects of work environmental air samples containing organic dust, endotoxins and microbial DNA in an in vitro model. Furthermore, blood leukocyte and plasma levels of general and lung-specific inflammatory biomarkers were studied in exposed waste workers in comparison to an unexposed control group. TLR activation patterns and biomarker expression were related to exposure levels and self-reported health effects, indicating that occupational exposure may.

### TLR activation potential of work environmental air samples

Occupational exposure to bioaerosols potentially triggers an immune response in susceptible individuals. However, due to the complexity of work environmental air, the effects of occupational exposure on workers’ health are difficult to study. In vitro assays provide an adequate option to investigate potential exposure-related immunological effects and dose–response relationships in a simplified model system. The present study utilized TLR-transfected HEK cell lines to study the potential of work environmental air samples to activate cellular signalling pathways that result in NFkB-mediated transcription of inflammatory genes. Significant activation of TLR2 and TLR4 was measured in about one-third of all samples (Fig. 2), of these the majority were collected at plant B, C and F. However, no obvious reasons that explain these trends could be identified. Furthermore, significant and strong correlations between TLR activation to total dust levels, endotoxins and microbial DNA concentrations were found (Fig. 3). These results confirm findings from previous studies in which significant activation of TLR was observed in work environmental samples from waste sorting (Afanou et al. 2023; Eriksen et al. 2022). As TLR2 and TLR4 stimulation is specifically initiated by microbial products, such as LPS and LTA, it can be assumed that the work-air samples contained bacterial ligands with pathophysiological potential. In vivo TLR activation of tissue resident and/or circulating immune cells may be triggered directly by microbial epitopes, or indirectly by interaction with secondary helper molecules (Liu et al. 2014). TLR signalling includes a wide array of intracellular regulatory pathways, such as the NFkB pathway, which mediates the transcription of regulatory genes that are involved in synthesizing inflammatory molecules, such as cytokines, chemokines and endothelial adhesion molecules (Ghosh et al. 2006). However, as work air samples contain a heterogenous mixture of bioaerosols, chemicals and other inhalable particles, it remains unclear which molecules contribute directly or as mediators to activating TLR-induced signalling pathways. In this study, activation of TLR2 and TLR4-induced signalling pathways was measured as SEAP levels in the cell supernatant after exposure, providing a measurement for the total activation potential of the air samples, rather than allowing the identification of distinctive molecules with toxicological capabilities. Repeated exposure in mouse model, however, showed an upregulation of pro-inflammatory cytokines, such as TNF-α, IL-17, IL-6 and IL1b and chemokines such as CCL2, as well as increased neutrophil recruitment to the lung tissue through TLR4-mediated signalling (Cui et al. 2020). These results emphasize the relevance of model systems in occupational exposure research to investigate mechanisms of potential exposure-related health effects.

Results from in vitro experiments cannot fully explain exposure-related health outcomes in workers, as individual susceptibility naturally varies due to genetic polymorphisms, underlying health conditions, age, nicotine use, BMI, or external factors, such as long-term exposure to weak electro-magnetic fields (Hosseinabadi et al. 2019; Plummer et al. 2012; Schwartz and Cook 2005). However, based on the in vitro results in the present study, it can be assumed that occupational exposure during waste handling may elicit an immune response in susceptible workers.

### Levels of blood leukocytes and plasma biomarkers

The present study measured plasma levels of various biomarkers as well as blood leukocytes to investigate differences in exposure groups and day-to-day variation and to identify the biomarkers’ potential as exposure-related bioindicators. Furthermore, differences in biomarker levels between waste sorting plants were investigated; however, no consistent trends could be identified. Some of the assessed biomarkers have previously been proposed as potential bioindicators for respiratory function in vivo in an occupational setting (Bassig et al. 2013; Nakanishi et al. 2021; Smit et al. 2009). The majority of the biomarkers that were assessed in the present study were below the limit of detection. Among measurable analytes, however, leukocyte and biomarker levels were in general independent of exposure group and time of sample collection. This may be explained by the ephemerality of cytokines in the blood system, which exert their effect within hours of secretion (Liu et al. 2021). Thus, collecting samples before shift may have not captured the biomarkers during their biologically active period.

The present study showed significantly increased monocytic activity in exposed workers, especially on the first workday of the week in comparison to unexposed controls (Table 3, Figure S3). These results concord with previous studies that successfully correlated elevated blood leucocyte levels to occupational exposure (Ray et al. 2005; Salih et al. 2021). Wikuats et al. (2022) reported significantly lower monocyte levels among the exposed population in a recent study that investigated occupational exposure-related biomarker levels in waste workers. However, in this study the authors were not able to correlate biomarker levels to resulting health effects. As monocytes are one of the major sources of pro-inflammatory cytokines, such as interleukins and TNFs during an early immune response it can be assumed, that the observed increase in monocytes in the present study in association with significantly increased levels of IL-1Ra, IL-18 and TNFα can be related to occupational exposure. Pro-inflammatory cytokines, such as TNF-α and IL-18, are potent mediators of acute inflammation in response to microbial infections eliciting local and systemic inflammatory effects and facilitating a Th1-directed immune response (Iwamoto et al. 2007; Scheller et al. 2011). Both IL-18 and TNFα have been proposed as potential diagnostic and prognostic biomarker for the pathogenesis of respiratory disease (Mateu-Jimenez et al. 2017; Nakanishi et al. 2021). However, the value of these biomarkers as indicators for acute exposure-related health effects remains largely unknown. In contrast to IL-18 and TNFα, the anti-inflammatory interleukin 1 antagonist IL-1Ra acts as natural inhibitor of IL-1 signalling which is immediately upregulated upon encountering epitopes with toxicological properties (Akash et al. 2013). IL-1Ra levels in the present study were generally higher, though not significant, in exposed workers and tendentially higher midweek compared to Monday levels (Table S2, Figure S1, Table S4). This indicates that IL-1Ra secretion in exposed workers is upregulated in response to an exposure initiated increased IL-1.

The present study showed tendencies of increased plasma biomarker levels among exposed workers, as well as positive and significant correlations between various pro-inflammatory cytokines (Fig. 3, Figure S1), indicating that an innate immune response can be expected in susceptible individuals. However, this cross-sectional study lacks timely spaced repeated measurements to investigate the full biological spectre of the assessed biomarkers.

### Biomarkers in waste workers and the impact of gender, BMI and nicotine habits

Sex, age and BMI have previously been reported to influence plasma cytokine levels (de Torres et al. 2011; Stapleton et al. 2010; Timpson et al. 2011; Wegner et al. 2017). The present study identified significant though low effects of BMI on increased leukocyte, neutrophil and eosinophil levels, as well as procalcitonin and CRP levels independent of exposure group. However, substantial differences between sexes were identified in various plasma biomarkers, with significantly increased procalcitonin, ICAM1 and CCL2 levels in females compared to male participants, whereas CRP levels were significantly reduced, respectively. Women have previously been reported to display stronger pro-inflammatory immune responses compared to men in general (Klein and Flanagan 2016) and when challenged with endotoxins in particular (Wegner et al. 2017). However, the number of female participants was limited during the present study; thus, these gender-based differences may be biased by large differences in participant numbers. Furthermore, significant associations between sex and BMI have been reported previously (Khera et al. 2009). Such interaction may also affect biomarker levels in the present study. Smoking habits affected all assessed blood leukocyte levels positively, as well as strongly and significantly impaired IL-18 levels, especially among exposed waste workers (Table 2), thereby confirming trends as previously reported by Prescott et al. (1997). The results in the present study indicate that nicotine consumption may have additive or synergetic effects on lung epithelial cells in combination with occupational exposure to bioaerosols.

### Bioaerosol exposure and its impact on plasma biomarkers and workers’ health

Among the assessed biomarkers, IL-8 may be of relevance in association to acute exposure, as it has relatively long half-life (up to several days) compared to other cytokines (Remick 2005). IL-8 is a chemotactic cytokine that is typically secreted by monocytes and macrophages in response to bacterial invasions during an early immune response and is involved in neutrophil recruitment and activation at the site of infection (Bickel 1993; Harada et al. 1994). The present study showed that an increase in midweek IL-8 levels was strongly, however, not significantly correlated to increasing total dust levels (Table S2), indicating that repeated exposure during waste handling may have a cumulative effect on IL-8 levels in exposed workers. Furthermore, the engagement of the workers’ immune system through high midweek endotoxin levels was shown in a significant increase ICAM1, indicating that an exposure-related immune response is activated by epitopes that results in expression of the glycoprotein that is crucial for endothelial–leukocyte interactions in the respiratory system. These results confirm findings in waste handlers exposed to bioaerosols published by Heldal et al. (2003b) in which the authors reported a significant increase in IL-8 in induced sputum between workdays. However, the substantial exposure-related decrease in TNFα and IL-18 levels in combination with a significant reduction in lymphocyte and eosinophil levels in exposed workers that was observed between workdays in the current study (Table 3 & Table S2) implies that an acute immune response is initiated upon exposure early in the week, however, levels out during the workweek. These results contradict exposure-related biomarker expression in waste workers reported in a previous study by Heldal et al. (2003a), in which the authors identified a significant increase in blood leukocyte and biomarker levels between workdays. The present study showed significant positive correlations between SP-D levels and various blood leukocyte levels. SP-D has been shown to play a dual role in the innate immune system by exhibiting anti- and pro-inflammatory properties depending on its molecular structure (Guo et al. 2008; Matalon et al. 2009). As pro-inflammatory agent, SP-D opsonises invading pathogens and stimulates ingestion by macrophages in pulmonary tissue, whereas as anti-inflammatory agent it directly inhibits T lymphocyte proliferation and downregulates IL-6 and TNF-a synthesis in activated macrophages (Barrow et al. 2015; Borron et al. 2002; Liu et al. 2015). Due to its high target specificity and direct interaction with invading particles in the respiratory system, SP-D has been suggested as potential biomarker for lung disease (Hoegh et al. 2010; Sorensen 2018; Winkler et al. 2011). The present study identified tendentially higher SP-D levels among exposed workers, especially on the first workday of the week (Table 2, Figure S1), indicating that opsonisation of potentially pathogenic particles is promoted already after short periods of occupational exposure.

Despite differing exposure intensities and differences in blood leukocyte and plasma biomarker levels between exposure groups, there were no significant differences in symptom frequencies between exposure groups identified. This may be due to a healthy worker effect. Workers who are not affected by the work environment will be retained in the workforce for prolonged periods, whereas workers who experience negative work exposure-related health effects are more likely to terminate their employment (Baillargeon 2001). Furthermore, even though about ^1^/_3_ of all personal air samples caused significant activation of TLRs in vitro and thereby provided proof for the presence of potentially harmful substances in the work environment, the workers did not suffer from any health effects that could be directly linked to work exposure. It is possible that exposed workers have been desensitised to potentially pathogenic agents as they regularly encounter them in the work environment. Furthermore, it is possible that the identified biomarker levels are regulated in such a way that they did not cause systemic acute health effects, however, may potentially contribute to exposure-related long-term effects later in life. Personal respiratory devices were readily used under waste sorting and maintenance during the Covid pandemic to minimise the risk of infection. This unusually frequent use of respiratory masks may have influenced the blood leukocytes and plasma biomarkers levels, indicating that the workers are adequately protected from inhalable pathogens.

### Caveats of the study

This study has some shortcomings. As all sampling was conducted during the Covid pandemic, access to waste sorting plants was restricted and recruitment rates were therefore relatively low. This explains the large difference in sample sizes between exposure groups. Due to the low sample size, it was not possible to assess differences in exposure between waste sorting plants and/or work task that might be of importance in terms of risk assessment and health promotion. Furthermore, it remains unclear to what extent the strict pandemic-related hygiene regimes and frequent use of personal respiratory devices in the respective waste sorting plants affected occupational exposure and consequently the results presented in this study. Additionally, the timepoint of blood sampling early in the morning may have avoided diurnal fluctuation, however, may not have captured most of the assessed biomarkers during their biologically active period. Further research is needed to identify exposure-related biomarker expression in waste workers.

## Conclusion

One-third of all personal inhalable work air samples caused significant activation of TLR transfected HEK cells, indicating that the work environmental dust contained microbial ligands capable of inducing an immune response in vitro. Furthermore, TLR activation patterns significantly and positively correlated with exposure levels of total dust, endotoxins and microbial genomic DNA concentrations in air samples. These results suggested that blood leukocytes as well as pro-inflammatory biomarkers might be upregulated in exposed waste workers. The present study revealed differences in blood monocytes and plasma biomarker levels between an exposed and unexposed control group. However, no differences in symptom frequencies were reported between exposure groups. This may indicate that the workers’ immune systems are not engaged to such an extent as to provoke short term clinical symptoms, or that workers are adequately protected by their respiratory equipment.

## Supplementary Information

Below is the link to the electronic supplementary material.

Supplementary file1 (DOCX 310 KB)

## Data Availability

The datasets generated during and/or analysed during the current study are available from the corresponding author on reasonable request.

## Abbreviations

CCL2: Chemokine (C–C motif) ligand 2
CD40: Cluster of differentiation 40
DMEM: Dulbecco's Modified Eagle Medium
FBS: Foetal bovine serum
GM-CFS: Granulocyte–macrophage colony-stimulating factor
HEK: Human embryonic kidney cells
ICAM: Intercellular adhesion molecule
IFN: Interferon
IL: Interleukins
LPS: Lipopolysaccharide
LTA: Lipoteichoic acid
MMP-12: Matrix metalloproteinase-12
NFkB: Nuclear factor kappa B
PAMP: Pathogen-associated molecular patterns
PBS-BSA: Phosphate-Buffered Saline–bovine serum albumin
SP-D: Surfactant protein D
TLR: Toll-like receptors
TNF: Tumour necrosis factor
