# Supplementary material for: Bioaerosol-induced in vitro activation of toll-like receptors and inflammatory biomarker expression in waste workers
Source: Int Arch Occup Environ Health. 2023 May 27;96(7):985–98. doi: 10.1007/s00420-023-01984-7 (PMC10361871; doi:10.1007/s00420-023-01984-7)
Supplement: Supplementary file 1 — Supplementary file1 (DOCX 310 KB) [file 420_2023_1984_MOESM1_ESM.docx]

# Paper supplement A

Table S1 - Exposure measurements. The LOD for endotoxins analysed with LAL was 2.5 EU/filter. The LOD for total dust filter samples was 0.01 mg/m^3^. The identification threshold for positively loaded ddPCR droplets was set at 5000 for fungi and 9000 for bacteria.

|  | **n** | **AM** | **range** | **GM** | **GMsd** |
| --- | --- | --- | --- | --- | --- |
| **endotoxins** (EU/m^3^) | 116 | 219 | <LOD - 7479 | 47 | 5 |
| **total** **dust** (mg/m^3^) | 65 | 0.92 | 0.036 - 17.5 | 0.39 | 3.33 |
| **bacterial** **DNA** (copies/m^3^) | 112 | 2.2E+06 | 75 – 4.8E+07 | 1.1E+05 | 2.2E+01 |
| **fungal DNA** (copies/m^3^) | 112 | 1.1E+05 | 5 – 9.7E+05 | 1.2E+04 | 1.1E+01 |

Table S2 - Correlation of biological biomarkers (midweek levels) and Tuesday’s exposure levels of total dust, endotoxins, and bacterial and fungal DNA copies per m^3^. The linear model accounts for BMI, age, sex and smoking habits. All values are on a logarithmic scale. Exposed workers only.

|  | **endotoxin levels**  **n = 36** | | **total dust levels**  **n = 21** | | **bacterial DNA level**  **n = 36** | | **fungal DNA levels**  **n = 36** | |
| --- | --- | --- | --- | --- | --- | --- | --- | --- |
|  | Estimate | p value | Estimate | p value | Estimate | p value | Estimate | p value |
| **leukocytes** | -0.021 | 0.14 | -0.049 | 0.12 | -0.00077 | 0.92 | 0.0012 | 0.89 |
| **neutrophils** | -0.0029 | 0.90 | -0.051 | 0.29 | 0.0093 | 0.49 | 0.016 | 0.25 |
| **monocytes** | -0.045 | 0.096 | -0.065 | 0.26 | -0.014 | 0.36 | -0.0037 | 0.82 |
| **eosinophils** | -0.0065 | 0.49 | 0.012 | 0.48 | -0.003 | 0.59 | -0.0025 | 0.64 |
| **basophils** | -0.005 | 0.39 | ***-0.017*** | ***0.026*** | -0.00033 | 0.91 | -0.0013 | 0.67 |
| **CRP** | -0.10 | 0.23 | -0.14 | 0.29 | 0.026 | 0.57 | 0.051 | 0.33 |
| **IL-8** | 0.21 | 0.17 | *0.48* | *0.099* | -0.014 | 0.88 | 0.041 | 0.67 |
| **IL-18** | -0.098 | 0.049 | *-0.13* | *0.087* | -0.034 | 0.24 | 0.0016 | 0.99 |
| **IL-1Ra** | -0.13 | 0.41 | -0.18 | 0.46 | -0.087 | 0.35 | -0.031 | 0.32 |
| **TNF α** | ***-0.46*** | ***0.034*** | ***-0.88*** | ***0.010*** | -0.13 | 0.29 | -0.043 | 0.67 |
| **CCL2** | -0.009 | 0.83 | 0.015 | 0.87 | -0.0072 | 0.77 | -0.026 | 0.31 |
| **ICAM1** | ***0.17*** | ***0.066*** | 0.11 | 0.29 | 0.059 | 0.077 | 0.050 | 0.17 |
| **S100B** | -0.06 | 0.75 | -0.21 | 0.47 | 0.044 | 0.68 | 0.063 | 0.59 |
| **PCT** | 0.045 | 0.28 | 0.083 | 0.31 | 0.007 | 0.74 | 0.033 | 0.20 |
| **SP-D** | 0.024 | 0.52 | 0.079 | 0.15 | 0.0037 | 0.87 | -0.0053 | 0.83 |

Table S3 - Number of plasma samples analyses with LUMINEX by study group, day and sex.

| n total = 135 | control | exposed |
| --- | --- | --- |
| Monday / male | 16 | 48 |
| Monday / female | 5* | 3 |
| Wednesday / male | 13 | 43 |
| Wednesday / female | 4* | 3 |
| ** Samples of one female participant in the control group were removed from the analyses, as the person was self-reportedly ill on both sampling days.* | | |


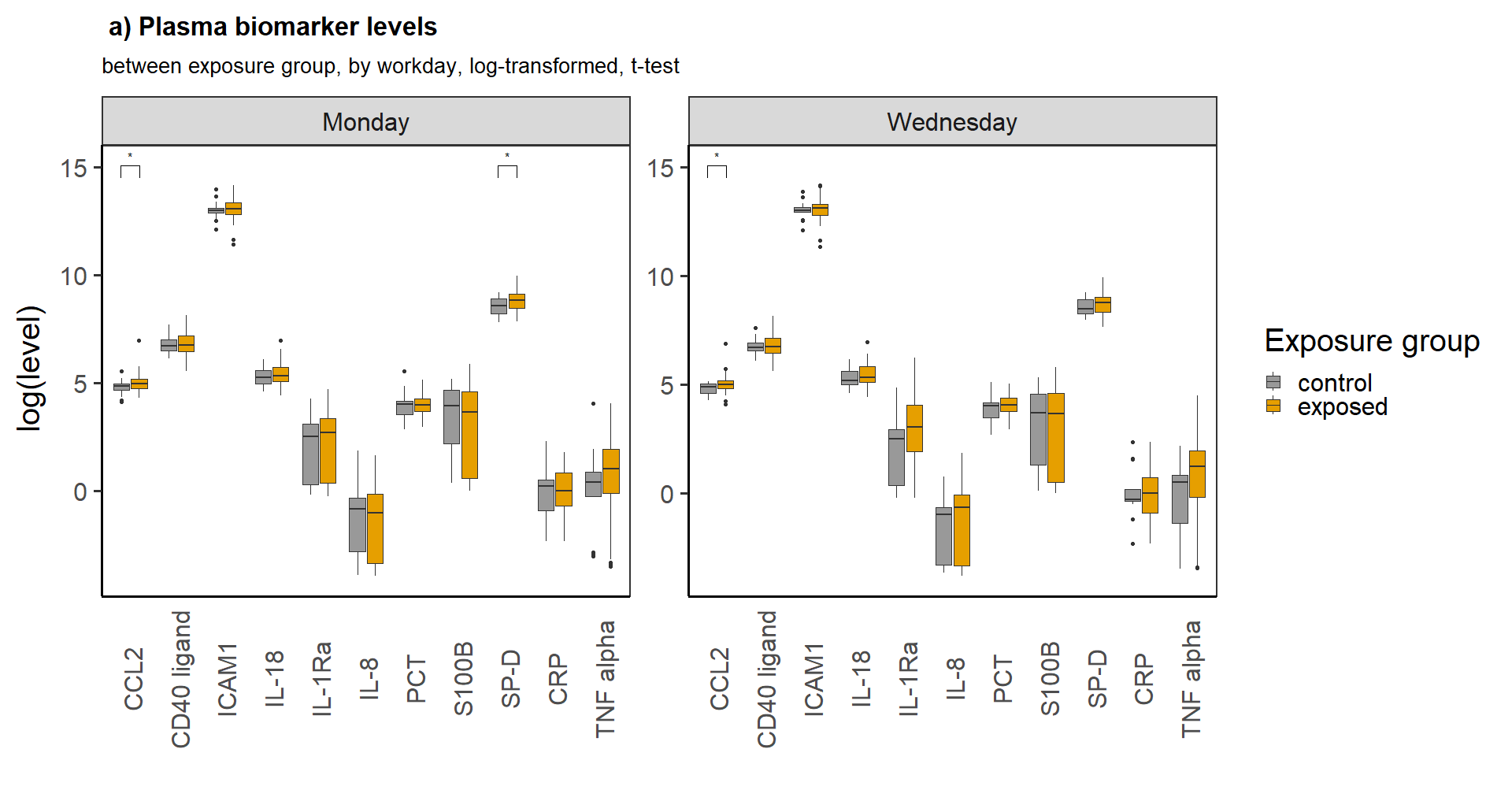


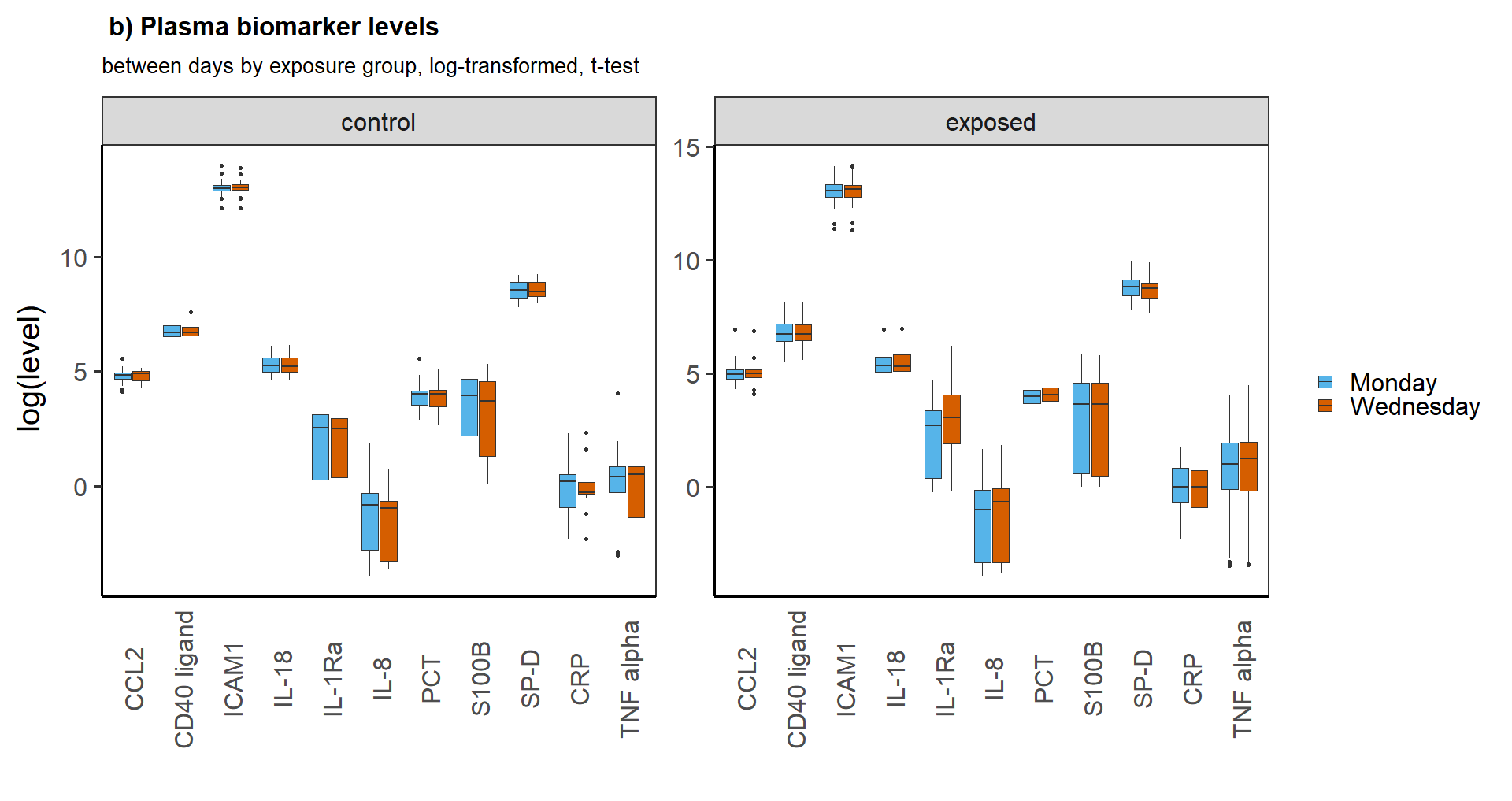


Figure S1 – Plasma biomarker levels (log pg mL^-1^) in comparison between exposed and controls by day. Upper panel (a) variation between exposure groups by workday, lower panel (b) variation between days within exposure group.


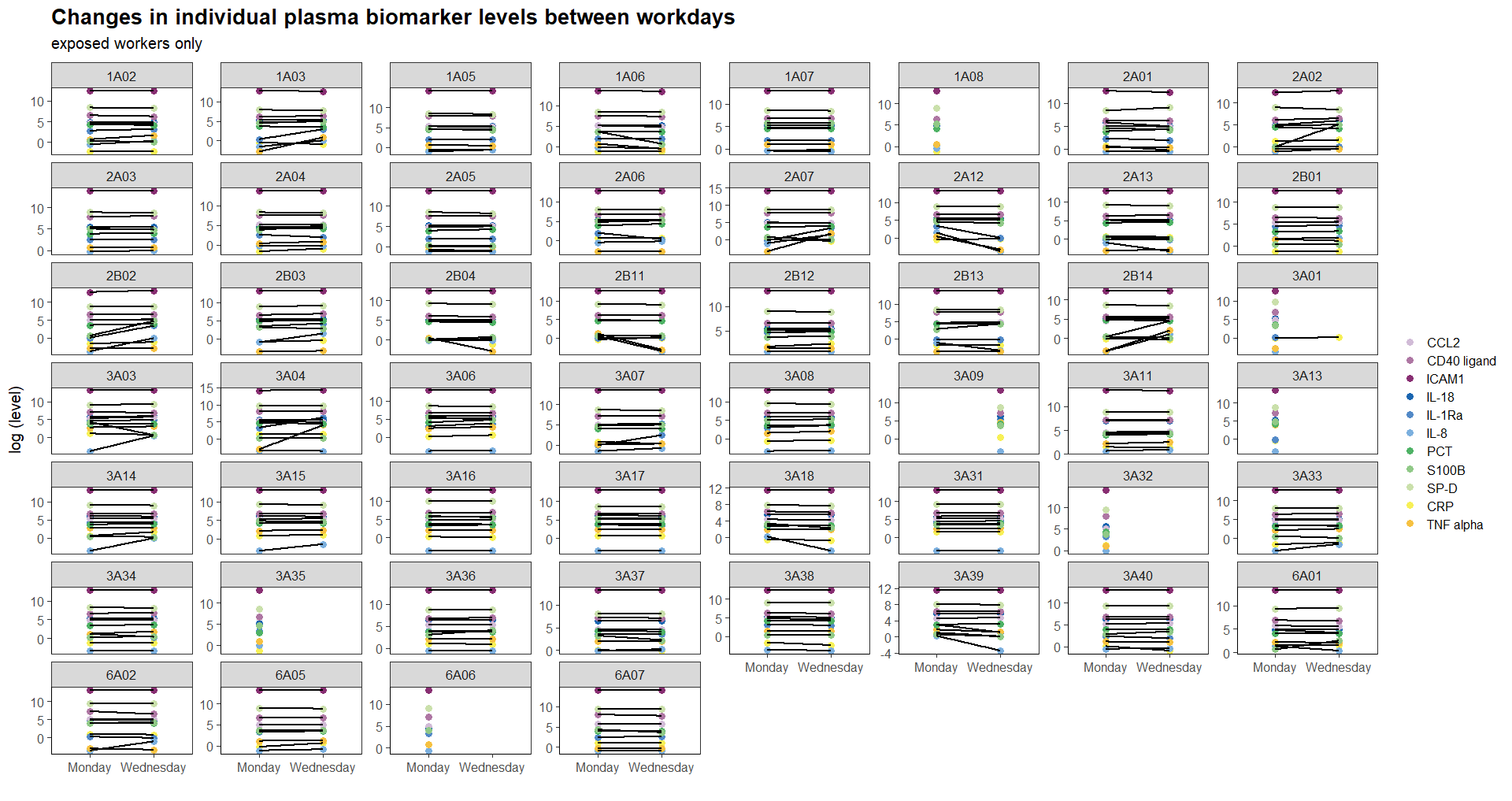


Figure S2 - Individual change of biomarker levels between days in exposed waste workers.

Table S4 – Changes in plasma biomarkers between days. Increase/decrease between Monday and Wednesday by exposed and control group.

|  | **exposed** | | | | **controls** | | | |
| --- | --- | --- | --- | --- | --- | --- | --- | --- |
|  | **n()** | **Increase (%)** | **Decrease (%)** | **no change (%)** | **n()** | **Increase (%)** | **Decrease (%)** | **no change (%)** |
| CCL2 | 44 | 23(52%) | 20 (45%) | 1 (2%) | 16 | 7 (44%) | 9 (56%) | 0 (0%) |
| CD40L | 44 | 23 (52%) | 20 (45%) | 1 (2%) | 16 | 9 (56%) | 6 (37%) | 1 (6%) |
| ICAM1 | 44 | 16 (37%) | 26 (59%) | 2 (5%) | 16 | 7 (44%) | 9 (56%) | 0 (0%) |
| IL-1Ra | 44 | 25 (59%) | 13 (30%) | 6 (13%) | 16 | 7 (44%) | 4 (25%) | 5 (31%) |
| IL-8 | 44 | 25 (59%) | 13 (30%) | 6 (13%) | 16 | 5 (31%) | 9 (56%) | 2 (12%) |
| IL-18 | 44 | 13 (30%) | 29 (66%) | 2 (5%) | 16 | 6 (37%) | 9 (56%) | 1 (6%) |
| PCT | 44 | 28 (64%) | 15 (34%) | 1 (2%) | 16 | 5 (31%) | 11 (69%) | 0 (0%) |
| S100B | 44 | 20 (45%) | 21 (47%) | 3 (7%) | 16 | 8 (50%) | 8 (50%) | 0 (0%) |
| SP-D | 44 | 12 (27%) | 32 (72%) | 0 (0%) | 16 | 9 (56%) | 7 (44%) | 0 (0%) |
| TNF-α | 44 | 20 (45%) | 16 (36%) | 8 (18%) | 16 | 7 (44%) | 7 (44%) | 2 (12%) |
| CRP | 44 | 18 (41%) | 19 (43%) | 7 (16%) | 17 | 2 (12%) | 14 (82%) | 1 (6%) |

Table S5 - Blood leukocyte levels outside the laboratories reference range (rr), by exposure group.

|  | **exposed** | | | | **controls** | | | |
| --- | --- | --- | --- | --- | --- | --- | --- | --- |
|  | **Monday** | | **Wednesday** | | **Monday** | | **Wednesday** | |
|  | **n ()** | **above/below rr** | **n ()** | **above/below rr** | **n ()** | **above rr** | **n ()** | **above/below rr** |
| **total** | **53** | **49** |  |  | **21** |  | **17** |  |
| B leukocytes (rr: 3.5 – 10.0) | 51 | 4 /1 | 47 | 1/1 | 21 | 1/0 | 17 | 1/0 |
| neutrophils (rr: 1.5 – 7.3) | 51 | 1/2 | 40 | 1/1 | 21 | 0/0 | 16 | 1/0 |
| lymphocytes (rr: 1.1 – 3.3) | 51 | 4/0 | 40 | 2/0 | 21 | 1/0 | 16 | 0/0 |
| monocytes (rr: 0.2 – 0.8) | 51 | 7/0 | 40 | 4/0 | 21 | 1/0 | 16 | 1/0 |
| eosinophils (rr: < 0.4) | 51 | 3 | 40 | 1 | 21 | 1 | 16 | 0 |
| basophils (rr: < 0.2) | 51 | 0 | 40 | 0 | 21 | 0 | 16 | 1 |
| CRP (rr: < 5 mg/L) | 49 | 1 | 47 | 1 | 21 | 2 | 17 | 0 |

Table S6 – Changes in individual blood leukocyte levels between days. Increase/decrease between Monday and Wednesday for exposed workers and control group.

|  | **exposed** | | | | **controls** | | | |
| --- | --- | --- | --- | --- | --- | --- | --- | --- |
|  | **n()** | **increase (%)** | **decrease (%)** | **no change (%)** | **n()** | **increase (%)** | **decrease (%)** | **no change (%)** |
| leukocytes | 46 | 17 (37%) | 28 (61%) | 1 (2%) | 17 | 12 (71%) | 5 (29%) | 0 (0%) |
| basophils | 39 | 4 (10%) | 1 (3%) | 34 (87%) | 16 | 3 (19%) | 1 (6%) | 12 (75%) |
| eosinophils | 39 | 5 (13%) | 8 (21%) | 26 (67%) | 16 | 1 (6%) | 5 (31%) | 10 (63%) |
| lymphocytes | 39 | 14 (36%) | 20 (51%) | 5 (13%) | 16 | 7 (44%) | 8 (50%) | 1 (6%) |
| monocytes | 39 | 11 (28%) | 20 (51%) | 8 (21%) | 16 | 7 (44%) | 6 (38%) | 3 (19%) |
| neutrophils | 39 | 18 (46%) | 20 (51%) | 1 (3%) | 16 | 11 (69%) | 5 (31%) | 0 (0%) |


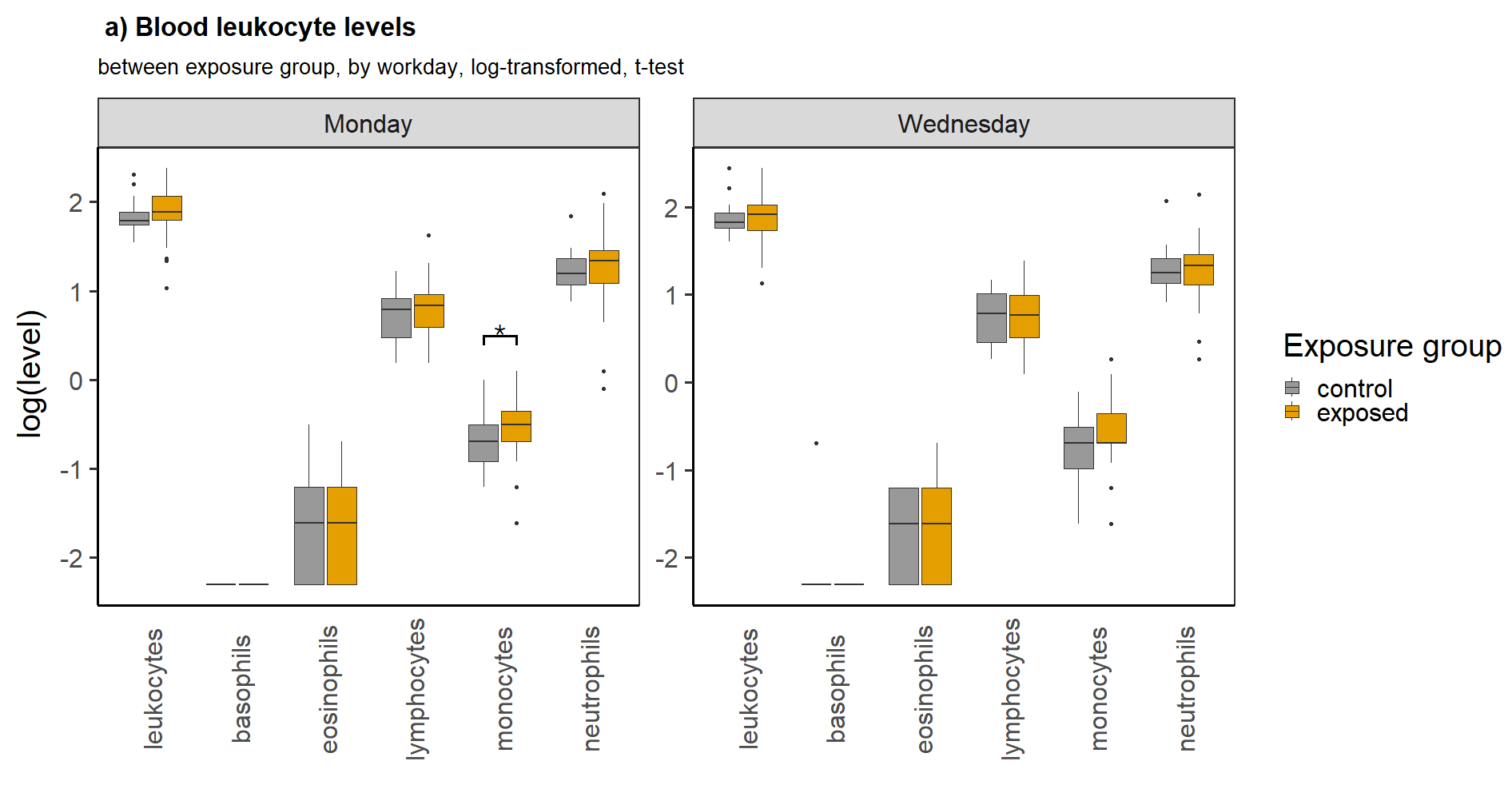


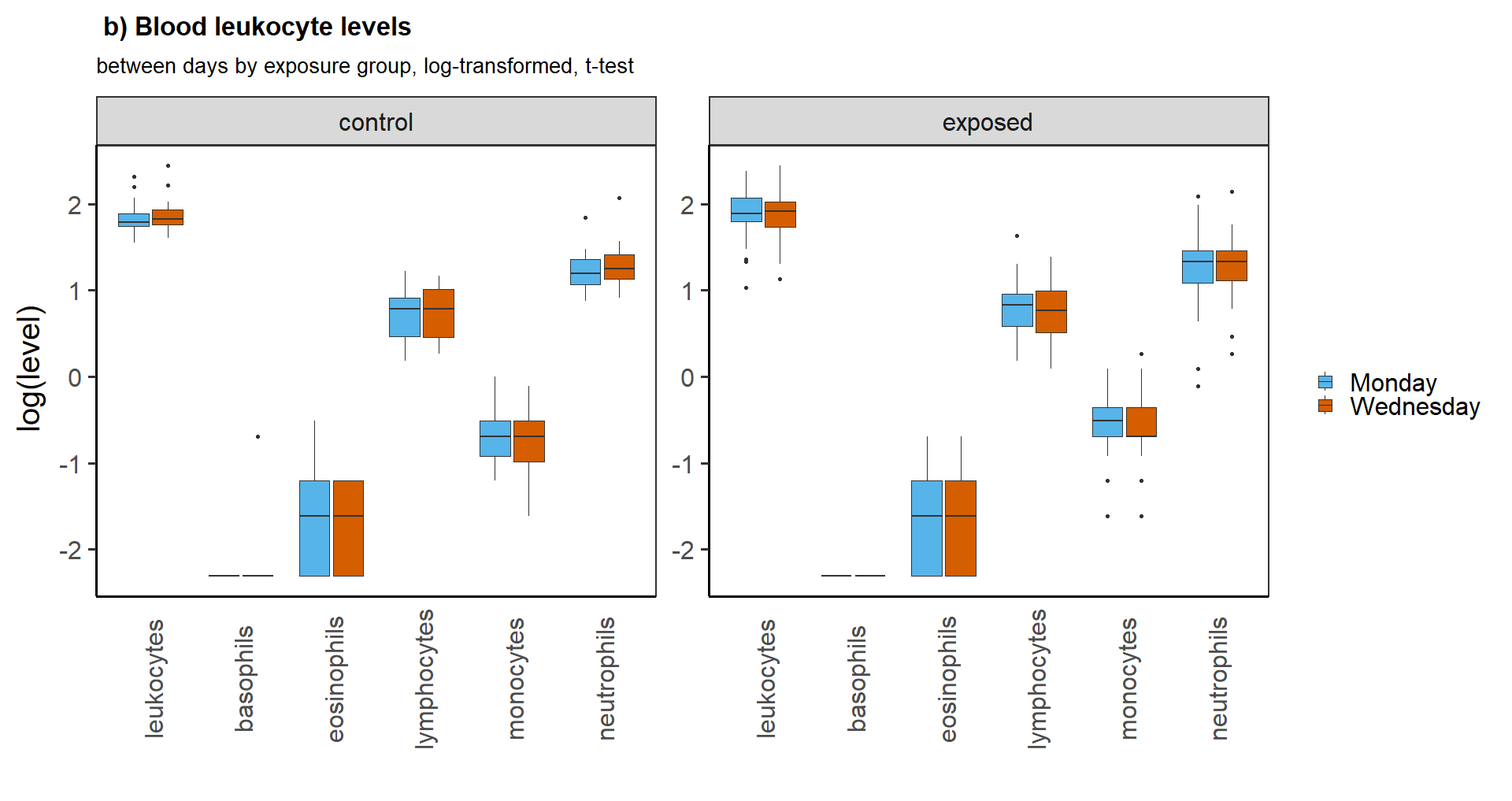


Figure S3 - Differences in mean blood leukocyte levels between groups and days. Upper panel (a) variation between exposure groups by workday, lower panel (b) variation between workdays by exposure group.


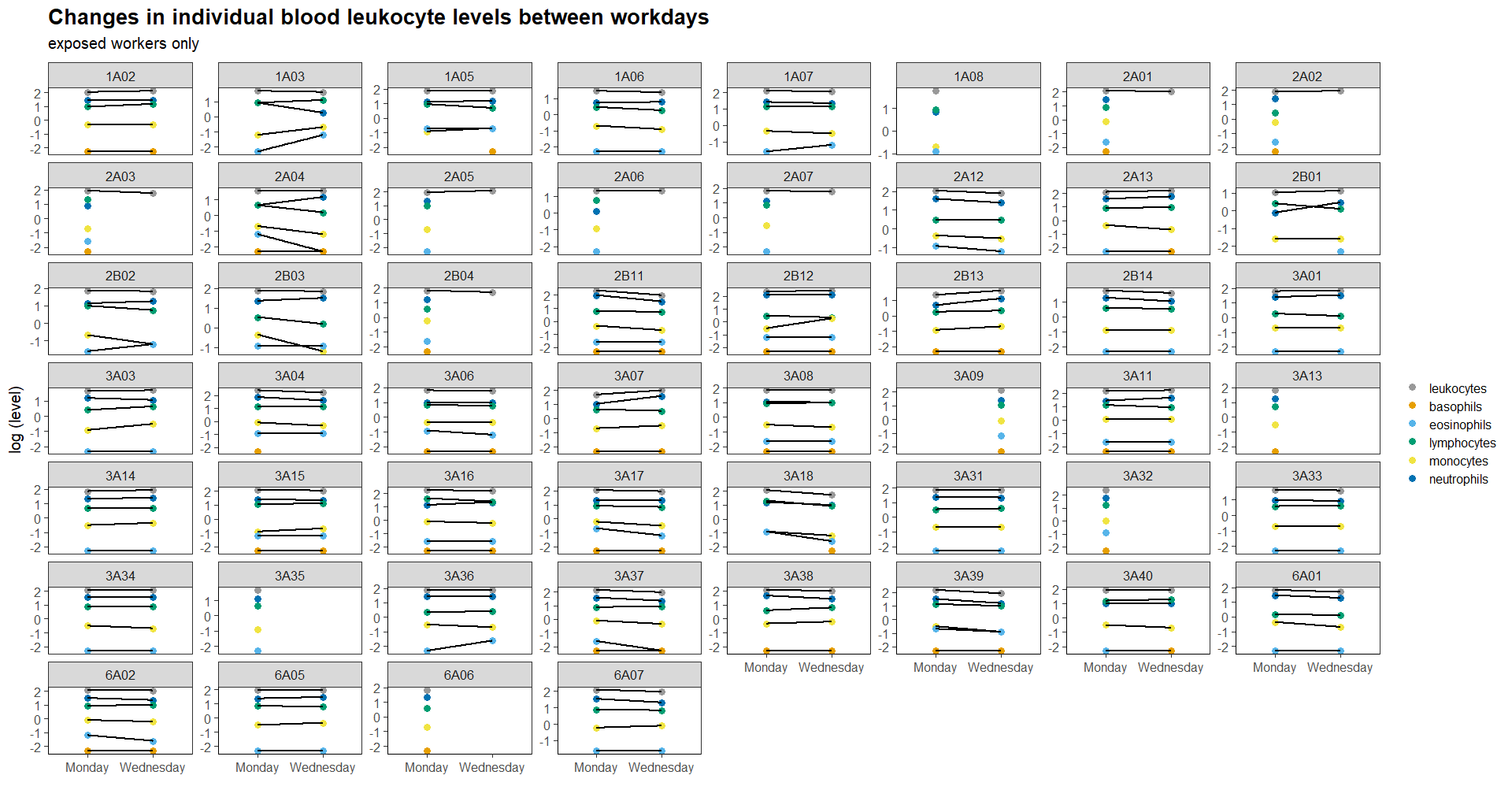


Figure S4 – Changes in individual blood leukocyte levels in exposed workers between workdays.

Table S7 - Correlation matrix blood leukocytes and plasma biomarkers in exposed workers. Upper half shows Pearson correlation coefficients, pair-wise correlation. Lower half shows corresponding Benjamini Hochberg adjusted p-values, significance level 0.05.

| **leukocytes** | 8.56E-01 | 5.33E-01 | 7.26E-01 | 3.73E-01 | 4.01E-01 | 3.68E-01 | -1.08E-01 | 4.37E-02 | 1.12E-01 | -1.53E-01 | 5.53E-02 | 3.23E-01 | 7.17E-02 | 3.05E-02 | 3.22E-01 | 2.90E-01 |
| --- | --- | --- | --- | --- | --- | --- | --- | --- | --- | --- | --- | --- | --- | --- | --- | --- |
| **3.16E-27** | **neutrophils** | 6.37E-02 | 5.91E-01 | 4.32E-01 | 2.09E-01 | 2.37E-01 | -6.93E-02 | 4.30E-02 | 1.49E-02 | -1.21E-01 | 1.10E-01 | 4.47E-01 | 3.14E-02 | -1.11E-01 | 3.11E-01 | 1.46E-01 |
| **5.50E-08** | 5.49E-01 | **lymphocytes** | 3.43E-01 | 8.37E-03 | 3.14E-01 | 2.80E-01 | -1.65E-01 | -8.96E-02 | 1.05E-01 | -1.58E-01 | -9.26E-02 | -1.53E-01 | 8.19E-02 | 1.84E-01 | 6.42E-02 | 3.11E-01 |
| **3.70E-16** | **6.97E-10** | **8.76E-04** | **monocytes** | 4.20E-01 | 2.14E-01 | 3.57E-01 | -1.82E-02 | 3.52E-02 | 1.28E-01 | -6.78E-02 | 2.14E-01 | 3.20E-01 | 1.77E-01 | 9.15E-02 | 3.36E-01 | 2.88E-01 |
| **1.83E-04** | **2.40E-05** | 9.38E-01 | **4.16E-05** | **Micro-CRP** | 1.71E-01 | 7.38E-02 | 6.28E-02 | 2.56E-01 | 1.46E-01 | 2.04E-01 | 1.77E-01 | 1.60E-01 | 1.43E-01 | 2.09E-01 | 3.79E-01 | 4.32E-01 |
| **8.25E-05** | **4.67E-02** | **2.44E-03** | **4.14E-02** | 1.09E-01 | **eosinophils** | 2.26E-01 | 2.98E-02 | 3.54E-02 | -4.44E-02 | -2.63E-02 | 6.03E-02 | 2.92E-01 | 1.57E-01 | 3.00E-01 | -8.69E-03 | 1.84E-01 |
| **3.34E-04** | **2.35E-02** | **7.13E-03** | **5.16E-04** | 4.92E-01 | **3.10E-02** | **basophils** | -1.41E-01 | -6.68E-02 | 9.23E-02 | -1.53E-01 | -6.07E-02 | 2.07E-01 | 7.38E-02 | 1.69E-01 | 3.12E-02 | 6.52E-02 |
| 2.92E-01 | 5.16E-01 | 1.21E-01 | 8.65E-01 | 5.46E-01 | 7.81E-01 | 1.85E-01 | **IL-8** | 2.42E-01 | 6.75E-02 | -4.98E-02 | 1.70E-01 | 1.57E-01 | 1.15E-01 | -5.28E-02 | -9.27E-02 | -4.14E-02 |
| 6.71E-01 | 6.87E-01 | 4.01E-01 | 7.42E-01 | **1.23E-02** | 7.41E-01 | 5.32E-01 | **1.69E-02** | **IL-1ra** | 6.89E-01 | 2.93E-01 | 5.52E-02 | -1.52E-01 | 9.53E-02 | 9.55E-02 | 1.59E-01 | 4.61E-01 |
| 2.76E-01 | 8.89E-01 | 3.26E-01 | 2.30E-01 | 1.58E-01 | 6.78E-01 | 3.87E-01 | 5.11E-01 | **5.88E-15** | **TNF-a** | 1.86E-01 | -1.40E-01 | -3.31E-01 | 2.48E-03 | 7.00E-02 | -4.32E-02 | 4.49E-01 |
| 1.35E-01 | 2.57E-01 | 1.37E-01 | 5.26E-01 | **4.77E-02** | 8.06E-01 | 1.50E-01 | 6.28E-01 | **3.53E-03** | 6.76E-02 | **CCL2** | 2.60E-01 | -9.01E-02 | 1.79E-01 | 1.81E-01 | 4.45E-01 | 1.56E-01 |
| 5.90E-01 | 3.02E-01 | 3.85E-01 | **4.24E-02** | 8.69E-02 | 5.72E-01 | 5.70E-01 | 9.62E-02 | 5.91E-01 | 1.73E-01 | **1.00E-02** | **ICAM1** | 3.01E-01 | 8.71E-01 | 2.23E-01 | 2.87E-01 | -2.57E-02 |
| **1.24E-03** | **1.02E-05** | 1.50E-01 | **2.08E-03** | 1.21E-01 | **5.17E-03** | 5.08E-02 | 1.25E-01 | 1.38E-01 | **9.40E-04** | 3.80E-01 | **2.73E-03** | **PCT** | 5.36E-02 | 6.48E-02 | 2.25E-01 | -1.05E-01 |
| 4.85E-01 | 7.69E-01 | 4.43E-01 | 9.50E-02 | 1.68E-01 | 1.39E-01 | 4.89E-01 | 2.64E-01 | 3.53E-01 | 9.81E-01 | 7.97E-02 | **4.57E-31** | 6.02E-01 | **CD40L** | 2.41E-01 | 1.71E-01 | 6.82E-02 |
| 7.67E-01 | 2.96E-01 | 8.27E-02 | 3.91E-01 | **4.24E-02** | **4.11E-03** | 1.10E-01 | 6.07E-01 | 3.52E-01 | 4.96E-01 | 7.59E-02 | **2.82E-02** | 5.28E-01 | **1.76E-02** | **S100B** | 3.46E-02 | 2.37E-01 |
| **1.30E-03** | **2.88E-03** | 5.48E-01 | **1.21E-03** | **1.55E-04** | 9.35E-01 | 7.71E-01 | 3.67E-01 | 1.20E-01 | 6.74E-01 | **5.00E-06** | **4.37E-03** | **2.69E-02** | 9.32E-02 | 7.36E-01 | **SP-D** | 1.12E-01 |
| **3.90E-03** | 1.70E-01 | **2.83E-03** | **5.90E-03** | **1.22E-05** | 8.25E-02 | 5.41E-01 | 6.88E-01 | **1.98E-06** | **4.08E-06** | 1.27E-01 | 8.03E-01 | 3.06E-01 | 5.07E-01 | **1.94E-02** | 2.75E-01 | **IL-18** |

Table S8 - Correlation matrix blood leukocytes and plasma biomarkers in control group. Upper half shows Pearson correlation coefficients, pair-wise correlation. Lower half shows corresponding Benjamini Hochberg adjusted p-values, significance level 0.05.

| **Leukocytes** | 8.42E-01 | 5.76E-01 | 5.48E-01 | 1.70E-01 | 4.74E-01 | 5.20E-01 | 1.77E-01 | 2.12E-01 | 1.80E-01 | -1.04E-01 | -1.35E-01 | -1.57E-01 | -1.11E-03 | -1.08E-02 | 8.59E-02 | 2.89E-01 |
| --- | --- | --- | --- | --- | --- | --- | --- | --- | --- | --- | --- | --- | --- | --- | --- | --- |
| 1.00E+00 | **neutrophils** | 6.85E-02 | 5.41E-01 | 8.67E-02 | 4.01E-01 | 5.47E-01 | -1.93E-02 | 1.43E-02 | 1.68E-01 | 3.95E-02 | -6.32E-02 | 1.21E-02 | 5.09E-02 | -5.78E-02 | 1.82E-01 | 4.37E-01 |
| **1.93E-04** | 6.87E-01 | **lymphocytes** | 4.03E-02 | 4.63E-02 | 1.38E-01 | 1.06E-01 | 3.56E-01 | 2.60E-01 | 1.35E-02 | -2.77E-01 | -8.79E-02 | -4.07E-01 | -4.50E-02 | 6.66E-03 | 2.79E-02 | -2.38E-01 |
| **4.47E-04** | **5.49E-04** | 8.13E-01 | **monocytes** | 1.22E-01 | 3.99E-01 | 2.56E-01 | 3.83E-01 | 1.37E-01 | 4.51E-01 | 1.52E-01 | -2.32E-01 | 4.57E-01 | -9.39E-02 | 1.76E-01 | -1.64E-01 | 3.98E-01 |
| 3.06E-01 | 6.10E-01 | 7.85E-01 | 4.71E-01 | **Micro**-**CRP** | 2.10E-01 | 3.04E-01 | 1.28E-02 | 5.25E-01 | 6.11E-02 | -6.81E-02 | 5.13E-02 | -4.70E-02 | -1.10E-01 | 2.26E-01 | 4.19E-02 | 1.97E-01 |
| **3.05E-03** | **1.38E-02** | 4.14E-01 | **1.44E-02** | 2.13E-01 | **eosinophils** | 3.29E-01 | 7.35E-02 | 2.70E-01 | 2.75E-01 | 1.28E-01 | -3.04E-01 | 1.29E-01 | -1.70E-01 | -7.96E-03 | -1.45E-01 | 5.76E-01 |
| **9.66E-04** | **4.63E-04** | 5.31E-01 | 1.27E-01 | 6.78E-02 | **4.70E-02** | **basophils** | 6.80E-02 | 3.32E-01 | 2.14E-01 | 1.18E-01 | -8.98E-02 | -1.34E-01 | 3.83E-02 | 2.10E-01 | 5.95E-02 | 3.90E-01 |
| 2.88E-01 | 9.10E-01 | **3.04E-02** | **1.94E-02** | 9.39E-01 | 6.65E-01 | 6.89E-01 | **IL_8** | 3.22E-01 | 5.32E-01 | 2.13E-02 | -1.24E-01 | 9.98E-02 | -1.51E-01 | 1.94E-01 | 1.25E-01 | -1.73E-01 |
| 2.01E-01 | 9.33E-01 | 1.21E-01 | 4.19E-01 | **7.22E-04** | 1.06E-01 | **4.50E-02** | **4.85E-02** | **IL_1ra** | 3.59E-01 | -1.05E-01 | -3.21E-02 | -8.66E-02 | 2.85E-02 | 3.53E-01 | -4.17E-02 | 3.64E-01 |
| 2.80E-01 | 3.21E-01 | 9.37E-01 | **5.13E-03** | 7.15E-01 | 9.93E-02 | 2.03E-01 | **5.89E-04** | **2.71E-02** | **TNF_a** | 2.71E-01 | 5.30E-03 | 3.56E-01 | -1.17E-02 | 2.50E-01 | -1.59E-01 | 3.39E-01 |
| 5.36E-01 | 8.16E-01 | 9.75E-02 | 3.68E-01 | 6.85E-01 | 4.52E-01 | 4.87E-01 | 8.99E-01 | 5.30E-01 | 9.93E-02 | **CCL2** | 2.87E-01 | 5.68E-01 | 2.71E-01 | 7.63E-02 | -4.01E-02 | 2.87E-01 |
| 4.17E-01 | 7.10E-01 | 6.05E-01 | 1.66E-01 | 7.60E-01 | 6.76E-02 | 5.97E-01 | 4.57E-01 | 8.48E-01 | 9.75E-01 | 8.02E-02 | **ICAM1** | 8.26E-02 | 8.29E-01 | -1.18E-01 | 1.08E-01 | -1.53E-01 |
| 3.47E-01 | 9.43E-01 | **1.25E-02** | **4.42E-03** | 7.79E-01 | 4.47E-01 | 4.29E-01 | 5.51E-01 | 6.05E-01 | **2.84E-02** | **2.02E-04** | 6.22E-01 | **PCT** | 1.23E-01 | -4.12E-02 | -2.12E-01 | 3.49E-01 |
| 9.95E-01 | 7.65E-01 | 7.92E-01 | 5.80E-01 | 5.09E-01 | 3.14E-01 | 8.22E-01 | 3.66E-01 | 8.65E-01 | 9.45E-01 | 1.00E-01 | **1.27E-10** | 4.61E-01 | **CD40L** | -1.07E-01 | -1.32E-01 | 6.27E-02 |
| 9.49E-01 | 7.34E-01 | 9.69E-01 | 2.97E-01 | 1.73E-01 | 9.63E-01 | 2.11E-01 | 2.43E-01 | **2.97E-02** | 1.30E-01 | 6.49E-01 | 4.81E-01 | 8.06E-01 | 5.21E-01 | **S100B** | -2.00E-01 | 2.54E-01 |
| 6.08E-01 | 2.82E-01 | 8.70E-01 | 3.31E-01 | 8.03E-01 | 3.93E-01 | 7.26E-01 | 4.54E-01 | 8.04E-01 | 3.39E-01 | 8.11E-01 | 5.20E-01 | 2.01E-01 | 4.29E-01 | 2.29E-01 | **SP_D** | -2.85E-01 |
| 7.89E-02 | **6.80E-03** | 1.55E-01 | **1.47E-02** | 2.37E-01 | **1.89E-04** | **1.69E-02** | 3.00E-01 | **2.48E-02** | **3.72E-02** | 8.09E-02 | 3.60E-01 | **3.19E-02** | 7.08E-01 | 1.23E-01 | 8.25E-02 | **IL_18** |

Table S9 - Standard reference values for LUMINEX bioplex analyses.

| **kit** | **ANALYTES** | **S1** | **S2** | **S3** | **S4** | **S5** | **S6** | **S7** | **S8** | **LOD** |
| --- | --- | --- | --- | --- | --- | --- | --- | --- | --- | --- |
| BioRad | **IFN-γ** | 18186 | 4546.50 | 1136.63 | 284.16 | 71.04 | 17.76 | 4.44 | 1.11 | 0.01 |
| BioRad | **IL-2** | 19223 | 4805.75 | 1201.44 | 300.36 | 75.09 | 18.77 | 4.69 | 1.17 | 0.03 |
| BioRad | **IL-4** | 5043 | 1260.75 | 315.19 | 78.80 | 19.70 | 4.92 | 1.23 | 0.31 | 0.01 |
| BioRad | **IL-6** | 5823 | 1455.75 | 363.94 | 90.98 | 22.75 | 5.69 | 1.42 | 0.36 | 0.02 |
| BioRad | **IL-8** | 15080 | 3770.00 | 942.50 | 235.63 | 58.91 | 14.73 | 3.68 | 0.92 | 0.02 |
| BioRad | **IL-10** | 17897 | 4474.25 | 1118.56 | 279.64 | 69.91 | 17.48 | 4.37 | 1.09 | 0.07 |
| BioRad | **GM-CSF** | 6881 | 1720.25 | 430.06 | 107.52 | 26.88 | 6.72 | 1.68 | 0.42 | 0.01 |
| BioRad | **TNF-α** | 62382 | 15595.50 | 3898.88 | 974.72 | 243.68 | 60.92 | 15.23 | 3.81 | 0.03 |
| BioRad | **IL-1β** | 4988 | 1247.00 | 311.75 | 77.94 | 19.48 | 4.87 | 1.22 | 0.30 | 0.30 |
| BioRad | **IL-12 (p70)** | 30652 | 7663.00 | 1915.75 | 478.94 | 119.73 | 29.93 | 7.48 | 1.87 | 0.02 |
| BioRad | **IL-1ra** | 153756 | 38439.00 | 9609.75 | 2402.44 | 600.61 | 150.15 | 37.54 | 9.38 | 0.70 |
| BioRad | **IL-1α** | 59639 | 14909.75 | 3727.44 | 931.86 | 232.96 | 58.24 | 14.56 | 3.64 | 0.18 |
| BioRad | **IL-13** | 3820 | 955.00 | 238.75 | 59.69 | 14.92 | 3.73 | 0.93 | 0.23 | 0.03 |
| R&D | **CCL2/JE/MCP-1** | 8680 | 2893.33 | 964.44 | 321.48 | 107.16 | 35.72 |  |  | 60 |
| R&D | **ICAM-1/CD54** | 877660 | 292553.33 | 97517.78 | 32505.93 | 10835.31 | 3611.77 |  |  | 84412 |
| R&D | **IL-2** | 8990 | 2996.67 | 998.89 | 332.96 | 110.99 | 37.00 |  |  | 1.81 |
| R&D | **IL-18/IL-1F4** | 1730 | 576.67 | 192.22 | 64.07 | 21.36 | 7.12 |  |  | 82 |
| R&D | **MMP-12** | 11390 | 3796.67 | 1265.56 | 421.85 | 140.62 | 46.87 |  |  | 1.12 |
| R&D | **S100B** | 13270 | 4423.33 | 1474.44 | 491.48 | 163.83 | 54.61 |  |  | 2.00 |
| R&D | **TGF-alpha** | 1450 | 483.33 | 161.11 | 53.70 | 17.90 | 5.97 |  |  | 0.22 |
| R&D | **CD40 ligand/TNFSF5** | 45090 | 15030.00 | 5010.00 | 1670.00 | 556.67 | 185.56 |  |  | 253 |
| R&D | **IL-1 beta/IL-1F2** | 4180 | 1393.33 | 464.44 | 154.81 | 51.60 | 17.20 |  |  | 0.53 |
| R&D | **IL-17/IL-17A** | 3240 | 1080.00 | 360.00 | 120.00 | 40.00 | 13.33 |  |  | 1.06 |
| R&D | **IL-33** | 3420 | 1140.00 | 380.00 | 126.67 | 42.22 | 14.07 |  |  | 0.31 |
| R&D | **Procalcitonin** | 1630 | 543.33 | 181.11 | 60.37 | 20.12 | 6.71 |  |  | 14.44 |
| R&D | **SP-D** | 55470 | 18490.00 | 6163.33 | 2054.44 | 684.81 | 228.27 |  |  | 2073 |
